# Membrane Trafficking of Death Receptors: Implications on Signalling

**DOI:** 10.3390/ijms140714475

**Published:** 2013-07-11

**Authors:** Wulf Schneider-Brachert, Ulrike Heigl, Martin Ehrenschwender

**Affiliations:** Institute for Clinical Microbiology and Hygiene, University of Regensburg, Franz-Josef-Strauss-Allee 11, Regensburg 93053, Germany; E-Mails: wulf.schneider@klinik.uni-regensburg.de (W.S.-B.); ulrike.heigl@klinik.uni-regensburg.de (U.H.)

**Keywords:** death receptor, apoptosis, endocytosis, membrane trafficking, receptor internalisation

## Abstract

Death receptors were initially recognised as potent inducers of apoptotic cell death and soon ambitious attempts were made to exploit selective ignition of controlled cellular suicide as therapeutic strategy in malignant diseases. However, the complexity of death receptor signalling has increased substantially during recent years. Beyond activation of the apoptotic cascade, involvement in a variety of cellular processes including inflammation, proliferation and immune response was recognised. Mechanistically, these findings raised the question how multipurpose receptors can ensure selective activation of a particular pathway. A growing body of evidence points to an elegant spatiotemporal regulation of composition and assembly of the receptor-associated signalling complex. Upon ligand binding, receptor recruitment in specialized membrane compartments, formation of receptor-ligand clusters and internalisation processes constitute key regulatory elements. In this review, we will summarise the current concepts of death receptor trafficking and its implications on receptor-associated signalling events.

## 1. Introduction

Molecules of the tumor necrosis factor (TNF) superfamily (TNFSF) and TNF-receptor superfamily (TNFRSF) are highly conserved and found in almost all mammalian cells. Evolutionarily, these proteins evolved with or soon after the divergence of bony fish and tetrapods, approximately 350–450 million years ago [1]. Receptor-ligand systems of this group are critically involved in various cellular signalling pathways such as inflammation, lymphocyte homeostasis, apoptosis and tissue development [2,3]. At the time of discovery, a subgroup of the TNFRSF, the so-called death receptors (DR), attracted considerable interest as robust cell-death induction upon ligand binding was recognised and triggered the ambitious goal for exploitation in therapeutic settings such as cancer therapy. However, especially during recent years it has become obvious that DR signalling is far more than cell death induction. Depending on individual (patho-) physiological circumstances, cell-type and involved receptor-ligand system, cellular responses range from activation of pro-inflammatory and potentially pro-tumoural pathways such as nuclear factor κB (NFκB), to caspase-dependent and -independent mechanisms of cell death induction. Concomitant with the awareness that DR signalling can be considered as a double-edged sword, the question how these multipurpose receptors ensure selective activation of a particular pathway raised. A number of elegant studies suggested a spatiotemporal regulation of signalling complex assembly and composition, with DR internalisation as a crucial event in this regulatory process. To date, this concept proved true for Tumor Necrosis Factor Receptor-1 (TNFR1), TNF-related apoptosis-inducing ligand receptor 1 (TRAIL-R1), TRAIL-R2, Fas and presumably also for death receptor 3 (DR3).

In this review, we will first briefly recapitulate general aspects and structural characteristics of DR signalling, before addressing pathways of specific receptor-ligand systems and their implications in (patho-) physiology in detail. Based thereon, we will discuss implications of membrane trafficking, especially receptor internalisation, on DR-associated signalling pathways.

## 2. Death Receptors: General Aspects and Essential Structural Characteristics

Structurally, TNFSF members are type-II transmembrane proteins, with a C-terminal TNF homology domain (THD). The latter is composed of 10 β-strands, folded to form a compact jellyroll-like topology [4]. The THD is weakly conserved (20%–30%) between TNFSF proteins. Most of the ligands are membrane-bound, but approximately half of them have proteolytic cleavage sites that allow conversion into a soluble form [5]. Interestingly, the membrane-bound and soluble forms may display strikingly different biological activities. Monomeric ligands are signalling-incompetent but gain biologic activity after homotrimer formation [2].

The cognate receptors are predominantly type-I transmembrane proteins, with only a few classified as type-III transmembrane proteins [2]. Comparable to TNF-ligands, TNF-receptors can also be converted into soluble molecules, either by shedding from the cell surface (often mediated by matrix-metalloproteases) or alternative mRNA splicing. In their extracellular part, molecules of the TNFRSF characteristically exhibit variable numbers of cysteine-rich domains (CRDs), which are critically involved in ligand binding. Presence of a protein-protein interaction platform in the intracellular domain, the so-called death domain, defines the “death receptor” (DR) subgroup of the TNFRSF [2]. Currently, the DR subgroup consists of TNFR1, Fas, DR3, TRAIL-R1, TRAIL-R2, death receptor 6 (DR6) and ectodysplasin A receptor (EDAR). The latter however is not considered as a “classical” DR as it is fails to induce caspase-dependent apoptosis and contains an atypical death domain (DD).

Structurally, the DD is a up-and-down antiparallel β-sheet fold with “Greek key” topology [6] that mediates homotypic interactions. Of note, DDs are not restricted to the intracellular part of DRs, but can also be found in proteins that are recruited to DRs. Additionally, topologically similar motifs, such as the death effector domain (DED), the caspase activation and recruitment domain (CARD) and the pyrin domain can be found in addition to or instead of a DD in proteins involved in DR signalling. Despite structural similarity, their surface charge is distinct and thereby confers specificity of association [7].

## 3. Death Receptor Signalling: A Double-Edged Sword

Among the TNFRSF, the signalling outcome may fundamentally differ between DR and non-DRs and is furthermore complicated by cross-talk with other, partly overlapping signalling pathways and membrane trafficking events that regulate or modulate assembly and/or association of the receptor signalling complex (RSC). Despite the suggestive designation “death receptor”, DR signalling is far more than cell death induction and also comprises non-apoptotic signalling pathways.

Binding of a trimeric ligand to the corresponding trimeric receptor triggers recruitment of intracellular adapter proteins [8]. The tri-fold design of receptor and ligand yields more contact sites and presumably achieves higher avidity [5]. For DRs, integrity of the intracellular death domain is essential for engaging downstream signalling components and initiation of both, apoptotic and non-apoptotic pathways. In a first step, DRs recruit (via homotypic DD interaction) a DD-containing adaptor molecule that can either be FADD (Fas-associated death domain) or TRADD (TNF-receptor associated death domain). In an early, simplistic model, the distinct recruitment of TRADD or FADD was considered of critical importance, as it was believed to set the course for DR signalling outcome. According to this concept, FADD-recruitment was assumed to be exclusively associated with apoptosis induction, while TRADD-recruitment enabled apoptotic and non-apoptotic signalling pathways. However, this postulation fell short as subsequent studies demonstrated engagement of non-apoptotic signalling pathways for “FADD-only” recruiting receptors such as Fas or TRAIL [9,10].

Both adaptor proteins in turn recruit distinct signalling complexes: TRADD recruits RIP1 (receptor-interacting serine/threonine-protein kinase 1) via the DD. Subsequently, RIP1 recruits TRAF2 (TNF-receptor associated factor 2), cIAP1 (cellular inhibitor of apoptosis protein 1) and cIAP2, triggering a downstream phosphorylation cascade finally culminating in activation of the inflammatory and survival pathways NFκB, JNK and p38 [11,12]. However, activation of non-apoptotic pathways is not exclusively triggered by TRADD-RIP1-TRAF2 complexes, as FADD-RIP1 and caspase-8 containing complexes are also capable to activate the NFκB pathway [9].

Non-apoptotic, TRADD-mediated DR-signalling is regulated by ubiquitination at various steps and is also a good example to demonstrate the impact of the specific type of the ubiquitin modification. Generally, monoubiquitination of proteins is believed to target them for endocytosis, whereas addition of K11- or K48-linked polyubiquitin chains is associated with proteasomal degradation. In contrast, K63-linked or linear polyubiquitin chains have roles in signal transduction and DNA repair [6].

Efficient activation of NFκB, JNK and p38 requires assembly of K63-linked polyubiquitin chains on RIP1, serving as docking sites for TAK1 (transforming growth factor-β activated kinase-1), TAB2 (TAK1 binding protein 2) and the regulatory subunit of the IκB kinase complex NEMO (NFκB essential modulator) [6]. The RIP1-associated proteins TRAF2 and cIAP1/2 both exhibit E3-ligase activity, and the individual contribution to the K63-linked polyubiquitination of RIP1 is still a matter of debate. Beside activating characteristics, shutdown of TRADD-mediated pro-inflammatory signalling also involves the ubiquitin system. Removal of K63-associated ubiquitin chains from RIP1 and replacement by K48-linked ubiquitin chains via the ubiquitin-editing enzyme A20 earmarks RIP1 for proteasomal degradation and thereby terminates NFκB signalling [13]. Ubiquitin-ligase activity of A20 itself is modulated by TAXBP1 (TAX binding protein 1) [14] and the ubiquitin ligase Itch [15]. Further complexity comes from additional RIP1 deubiquitination enzymes such as the A20-like protein Cezanne (cellular zinc finger anti-NFκB) [16] and CYLD [17]. For an in depth review of ubiquitination as regulatory mechanism of DR signalling, the reader is refered to [6].

In contrast to TRADD, the alternative adapter protein FADD possesses beside the DD an additional DED, a structurally similar protein-protein interaction motif. The DD of FADD allows direct recruitment to the DD of DRs, but also association with TRADD and therefore recruitment to initially TRADD binding receptors. In addition, the DED mediates interaction with caspase-8 and caspase-10 by homotypic interaction with the DED in the prodomain of these molecules [18].

In context of caspase-dependent cell death induction, signalling pathways emanating from TRADD or FADD binding DRs run together at the point of FADD oligomerisation that triggers caspase-8 and caspase-10 activation through an induced proximity change of conformation. The complex of a DR with FADD and caspase-8 or caspase-10 constitutes the death-inducing signalling complex (DISC). FADD and caspase-8 or caspase-10 can either be directly associated with DRs (FADD binding DRs), or through DD-mediated recruitment of the complex to TRADD or RIP1. Therefore, complexes containing FADD and caspase-8 or caspase-10, regardless whether directly assembled at the DD of DRs or recruited via TRADD or RIP1, can all be considered as DISCs.

Understandably, caspase activation is a tightly regulated process to avoid undesired cellular damage. At the DR level, DISC-mediated caspase activation is regulated by the long isoform of the cellular FLICE-like inhibitory protein (cFLIP-Long or cFLIP_L_). This molecule exhibits considerable sequence homology with caspase-8 and caspase-10, but lacks a crucial cysteine residue in the catalytic center and is enzymatically inactive. cFLIP_L_ prevents caspase-8 and caspase-10 activation by DED-mediated binding to FADD, thereby occupying the binding sites for caspases [19].

Ignition of the apoptotic cascade through caspase activation can be subdivided in an initiator phase, characterized by unlocking the initiator (or apical) caspase-8 and caspase-10. The execution phase of the apoptotic programme starts with proteolytic cleavage of the corresponding effector caspases (caspase-3, caspase-6, and caspase-7), which in turn again cleave essential cellular substrates and thereby execute the apoptotic cell death programme [18].

Mechanistically, the mode of transition from initiator to execution phase allows identification of two distinct cell types. In so-called type-I cells, activation of initiator caspases is sufficient for robust triggering of the death machinery and yields sufficient amounts of active effector caspases. In sharp contrast, activation of initiator caspases is insufficient in type-II cells. Insights in the biochemical basis came from recent studies, tracing back failure of robust caspase activation in type-II cells to low cell surface DR expression [20], low caspase expression levels and/or presence of caspase inhibitory molecules such as XIAP [21,22].

**Figure 1:**
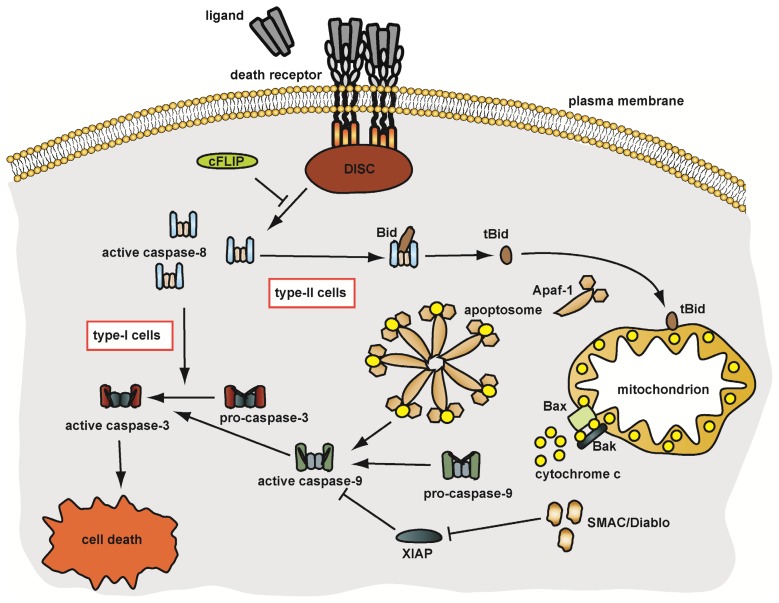
Apoptosis induction in type-I and type-II cells. See text for details.

Therefore, these cells require a mitochondrial amplification loop to efficiently activate effector caspases. In this scenario, caspase-8-mediated cleavage of the BH3-only protein Bid (BH3-interacting domain death agonist) generates truncated Bid (tBid), which translocates to the outer mitochondrial membrane (OMM) and allosterically activates Bak (Bcl-2 homologous antagonist/killer). Subsequent oligomerisation of Bak and Bax (Bcl-2 associated X-protein) forms pores in the OMM and allows escape of pro-apoptotic proteins such as cytochrome c, SMAC/DIABLO (second mitochondria-derived activator of caspase/direct IAP binding protein with low pI) and HtrA2/Omi into the cytoplasm [18,23–25]. Upon release, cytochrome c binds together with ATP to Apaf-1 (apoptosis promoting factor-1), forming the apoptosome which mediates caspase-9 activation [26]. Caspase-9 like caspase-8 is an initiator caspase, which in turn processes and activates caspase-3. Therefore, apoptotic pathways in type-I and type-II cells converge at the level of effector caspase activation. Figure 1 summarises apoptosis induction in type-I and type-II cells.

## 4. Structure, Physiology and Pathophysiology of TNFR1, TRAIL-R1/2, Fas and DR3

In this section, we address the specific characteristics of individual receptor-ligand systems of the DR group. Although membrane trafficking events have been recognised as key regulatory processes in DR signalling, not all members of the DR subgroup have been thoroughly characterised in this respect. In fact, data on the implications of membrane trafficking on death receptor signalling exist for TNFR1, TRAIL-R1, TRAIL-R2, Fas and partly for DR3. Therefore, we will focus on these members of the TNFRSF and leave other DRs such as EDAR and DR6 aside.

### 4.1. The TNF/TNFR1 System

#### 4.1.1. Structure, Physiological and Pathophysiological Roles of the TNF/TNFR1 System

##### 4.1.1.1. Structure

The TNF gene is located on chromosome 6p21.3. TNF is primarily produced as homotrimeric type-II transmembrane protein [27,28], but can be released via proteolytic cleavage by the metalloprotease TNF-alpha converting enzyme (TACE) [29]. Trimeric TNF appears at a molecular weight of 51 kDa, the 17 kDa protomers are composed of antiparallel β-strands folded into a compact, jellyroll-like topology and often referred to as the TNF homology domain (THD).

The gene for TNFR1 is located on chromosome 12p13.31, encoding a protein of 425 aa [30]. On SDS-PAGE, TNFR1 appears at a molecular weight of 55 kDa. The extracellular domain of TNFR1 has four cysteine-rich domains (CRDs). Ligand-binding occurs primarily between CDR2 and CRD3, whereas CRD1 has a role for TNFR1 multimerisation [31]. In the intracellular part of TNFR1, directly underneath the plasma membrane, the TNFR1 internalisation domain (TRID) is located with a tyrosine-based (YQRW) motif for clathrin-mediated internalisation [32]. Beside the death domain, the intracellular part of TNFR1 also harbours an interaction motif (aa 309–319) for the adaptor protein FAN (factor associated with neutral sphingomyelinase activation), which in turn binds and activates membrane-bound neutral sphingomyelinase (NSMase) [33].

##### 4.1.1.2. Physiological and Pathophysiological Roles of the TNF/TNFR1 System

A profound coverage of the pleiotropic TNF/TNFR1 system in health and disease would require a book rather than a review and is therefore beyond the scope. In brief, a role for TNF has been suggested in autoimmune diseases, such as psoriasis, inflammatory bowel disease, arthritis, systemic sclerosis, diabetes mellitus and multiple sclerosis [34]. In many of these pathologies, blockade of TNF signalling provided substantial benefit for the patients and significantly ameliorated clinical symptoms [35]. In cancer cells, TNF signalling is a mixed blessing as TNFR1 can trigger apoptotic cell death of tumour cells, but under most circumstances the signalling originating from TNFR1 is pro-inflammatory. This in turn is potentially tumourigenic, and TNF can indeed promote tumour growth, proliferation of tumour cells, as well as angiogenesis [36], invasiveness and metastasis [34]. In fact, TNF- and TNFR1-deficient mice developed fewer tumours after exposure to a skin carcinogen [37,38]. Additionally, TNFR1-deficiency attenuated experimental lung and liver metastasis in a mouse model [39,40] and treatment of tumour cells or mice with TNF increased the metastatic activity of transplanted malignant cells [41,42]. As wild-type mice whose bone marrow was repopulated with cells from TNFR1-deficient mice exhibited a reduced development of colitis and colon cancer [43], the tumour promoting properties of TNF were at least partly attributed to its action on TNFR1-positive myeloid cells resulting in cancer-related inflammation. Indeed, the TNF-driven inflammatory response in the tumour microenvironement (mainly mediated via the NFκB pathway) is associated with proliferation and survival of malignant cells and [44,45].

In addition to the involvement of TNF/TNF1 in autoimmune disease and malignancy, mouse models highlighted the role of this receptor ligand-system in the immune system. In particular, TNF or TNFR1 deficiency rendered these mice highly susceptible to the intracellular pathogen *Listeria monocytogenes* [46] or diminished clearance of *Mycobacterium tuberculosis* [47].

#### 4.1.2. Pathways of TNFR1 Signalling

During recent years, information on TNFR1 signalling virtually exploded and painted a complicated picture of signalling events associated with TNFR1. Especially, new regulatory mechanisms such as ubiquitination of signalling components attracted considerable attention [6,48].

Although TNFR1 can trigger the apoptotic cascade, under physiological conditions TNFR1 primarily exerts pro-inflammatory effects. Within minutes after ligand binding, RIP1, TRAF2 (TNF receptor-associated factor 2) and cIAP1/2 (inhibitor of apoptosis protein 1/2) are recruited to the receptor and form a complex often referred to as complex I [49,50]. The presence of TRADD in this conglomerate is controversially discussed [32,49]. Assembly of complex I requires translocation of TNFR1 in lipid rafts, where several proteins of this complex are post-translationally modified [51], e.g., K63 ubiquitination of RIP1 by the E3 ligases cIAP1 and cIAP2 at Lys377, an essential step for NFκB activation [52]. Ubiquitinated RIP1 mediates recruitment of TAK1 (transforming growth factor-beta-activated kinase 1) and IKK (Inhibitor of nuclear factor kappa-B kinase) to the TNFR1 complex [53], finally activating the pro-inflammatory NFκB pathway. However, TRAF2-RIP1 complexes are also capable of JNK (c-Jun N-terminal kinase) activation via MEKK1 (MAPK/ERK kinase kinase 1) and JNK kinase. JNK activation constitutes a prodeath stimulus, which is normally counteracted by NFκB-regulated expression of antiapoptotic proteins such as XIAP (X-linked inhibitor of apoptosis protein) [54]. However, sustained JNK activation results in phosphorylation and activation of the E3 ubiquitin ligase Itch. Itch-mediated ubiquitination of the antiapoptotic protein cFLIP promotes proteasomal degradation of the latter, thereby allows caspase-8 activation and shifts TNFR1 signalling from non-apoptotic to apoptosis-induction. After this initial complex formation at the plasma membrane, TNFR1 undergoes clathrin-mediated endocytosis [32], a decisive step for the outcome of TNFR1 signalling. Effects of TNFR1 internalisation are discussed in detail below.

#### 4.1.3. Membrane Trafficking and Implications on TNFR1 Signalling

Receptor internalisation is a crucial event in TNFR1 signalling and significantly influences the cellular response of this multipurpose receptor. Upon ligand binding, TNFR1 undergoes clathrin-dependent internalisation in various cell types. The first hints for a regulatory role of TNFR1 internalisation arose from studies with human endothelial cells, demonstrating a decrease of TNF-induced expression of NFκB target genes after blockade of receptor internalisation [55]. A subsequent study elegantly showed in U937 cells that TNFR1-associated activation of endo-lysosomal ASMase (acid sphingomyelinase), JNK and apoptosis induction were critically dependent on receptor internalisation and severely impaired after blocking clathrin-coated pit formation. Interestingly, activation of NSMase at the plasma membrane level was unaffected [56]. This indicated a role for TNFR1 compartimentalisation in selective signalling pathway activation: pro-apoptotic signalling emanated from intracellular vesicles and required receptor internalisation, whereas activation of non-apoptotic signalling pathways could start at the plasma membrane level and were shown to be internalisation-independent.

Indeed, propagation of the death signal from TNFR1-containing vesicles has been resolved at the molecular level. TNFR1 internalisation was essential for assembly of the DISC [32,57]: upon TNF stimulation, recruitment of TRADD, FADD and caspase-8 was detectable in Rab4 (Ras-related protein Rab-4A) and Rab5-positive TNFR1-receptosomes already after 3 minutes. Caspase-8 recruitment to TNFR1-containing vesicles was critically dependent on ALG-2 (apoptosis-linked gene 2 protein) and Alix (ALG-2 interacting protein X) [58]. Blockade of TNFR1 internalisation by disruption or deletion of the TNFR1 tyrosine-based internalisation motif (aa 207–210, YQRW motif) abrogated TRADD, FADD and caspase-8 recruitment and protected cells from TNFR1-induced apoptosis. In line with this, blockade of TNFR1 internalisation by the E3-14.7K protein in adenovirus infected cells was protective against TNFR1-induced apoptosis [57]. In sharp contrast, in both scenarios recruitment of RIP1 and activation of the NFκB pathway was unaffected [32]. Moreover, triggering the caspase cascade is not the only pro-apoptotic signal emanating from intracellular TNFR1-containing vesicles. Therein, TNFR1-mediated caspase-8 activation triggers proteolytic cleavage and activation of receptosome-associated caspase-7, which in turn cleaves and activates ASMase [59]. ASMase-mediated ceramide production generates active cathepsin D, which cleaves Bid into tBid [60] and thereby engages the mitochondrial amplification loop of apoptosis. In addition, TNFR1 internalisation might also trigger an alternative mechanism of ASMase activation involving reactive oxygen species. Interestingly, TNFR1 interacts with riboflavin kinase (RFK) and is hereby coupled to the ROS-generating NADPH oxidase enzymes Nox-1, Nox-2 and the common subunit p22phox [61]. Similar to caspase-7-mediated ASMase activation, ROS-mediated activation culminates in involvement of the apoptotic mitochondrial amplification loop.

Internalisation of TNFR1 does not only trigger pro-apoptotic signals, but also terminates NFκB activation that occurred at plasma membrane level. Two E3 ubiquitin ligases, CARP1 (cell division cycle and apoptosis regulator protein 1) and CARP2 colocalize with TNFR1 in the endosomal compartment and mediate RIP1 ubiquitination, targeting the molecule for proteasomal degradation [62].

Another membrane trafficking event involved in regulation of TNFR1 signalling preceeds receptor internalization. Upon ligand binding, TNFR1 translocation into membrane lipid rafts was identified as a prerequisite for association with TRADD, TRAF2 and RIP1. Additionally, ubiquitination of RIP1, a crucial event for TNFR1-mediated NFκB activation, was also reported to be dependent on lipid raft association of TNFR1 [51]. Consequently, interference with lipid raft organization blocked pro-inflammatory TNFR1 signalling and shifted the outcome towards apoptosis induction.

**Figure 2:**
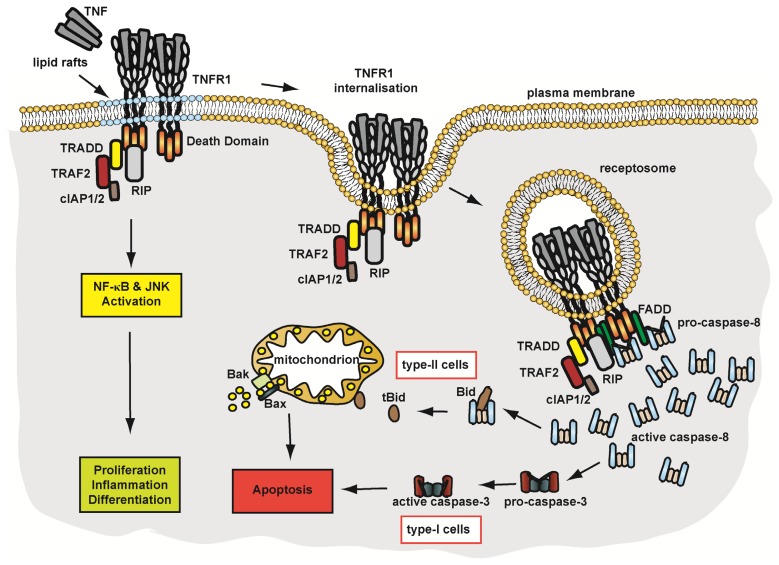
TNFR1-mediated signalling pathways. See text for details.

Taken together, TNFR1 internalisation actively shuts down pro-inflammatory and pro-survival NFκB signalling emanating from the plasma membrane, allows DISC assembly with subsequent caspase activation and furthermore activates ASMase potentially via a caspase-dependent and a caspase-independent mechanism. Therefore, trafficking of TNFR1 from the plasma membrane into TNFR1-receptosomes determines the signalling outcome of this pleiotropic receptor and is decisive for apoptotic or non-apoptotic TNFR1 signalling. A schematic overview of TNFR1 signalling is given in Figure 2.

### 4.2. The TRAIL/TRAIL-Receptor System

#### 4.2.1. Structure, Physiological and Pathophysiological Roles of the TRAIL/TRAIL-Receptor System

##### 4.2.1.1. Structure

The TNF-related apoptosis-inducing ligand (TRAIL) was discovered in 1995 by searching expressed sequence tag databases with a sequence from a conserved region of TNF family members [63]. The gene for TRAIL is located on chromosome 3q26, spans approximately 20 kb and contains five exons. Soon after discovery, it was recognised that the membrane-bound and an engineered soluble form triggered cell death in a wide variety of transformed cells.

TRAIL can bind to at least five different receptors: beside the two apoptosis-inducing receptors TRAIL-receptor-1 (TRAIL-R1, also DR4 or TNFRSF10A) and TRAIL-R2 (DR5 or TNFRSF10B), two additional so-called “decoy-receptors” TRAIL-R3 (DcR1) and TRAIL-R4 (DcR2) exist, both of which are incapable of transmitting apoptotic signals. Lastly, TRAIL can bind to the soluble osteoprotegerin receptor [64]. TRAIL-R1 and TRAIL-R2 were identified in 1997 [65,66] and both were mapped to chromosome 8p21.3. The extracellular domain of these receptors contains two complete CRDs, which are important for ligand binding. A recent report suggested a co-regulatory role for the transmembrane domain of TRAIL-R1 and TRAIL-R2 for the apoptotic signalling capacity [67]. The intracellular part of TRAIL-R1 and TRAIL-R2 harbours a full length death domain. TRAIL-R4 exhibits a truncated and non-functional DD in terms of apoptosis induction [64]. TRAIL-R3 is attached by a glycophosphatidylinositol anchor to the plasma membrane and has no intracellular domains at all. mRNA expression of all four TRAIL-receptors is found in virtually every tissue. Of note, this does not correlate with cell surface expression of the receptor(s), which is regarded as essential to fulfil physiological functions.

##### 4.2.1.2. Physiological and Pathophysiological Roles of the TRAIL/TRAIL-Receptor System

Knockout mice for the TRAIL-orthologue MK or the only murine TRAIL-receptor mDR5 [68] do not show an overt phenotype [69,70]. Initially, TRAIL-induced cell death induction was believed to be restricted to malignant cells [63], but subsequent studies also reported apoptosis induction in non-transformed cells [71]. However, exploitation of TRAIL or TRAIL receptor agonists for cancer therapy, either as single agents or in combination with conventional chemotherapy, is still a field of intensive research and recombinant TRAIL as well as agonistic, TRAIL-receptor targeting antibodies entered phase I and II trials [72]. However, as already outlined for the TNF/TNFR1 system, TRAIL-signalling in apoptosis resistant tumour cells can result in promotion of tumour metastasis and upregulation of pro-inflammatory factors. In the apoptosis resistant pancreatic ductal adenocarcinoma cell line Colo357, TRAIL treatment enhanced expression of pro-inflammatory cytokines such as interleukin-8 and monocyte chemoattractant protein 1 (MCP1) and aggravated tumour cell invasion by upregulation of urokinase-type plasminogen activator expression *in vitro* and in a orthotopic metastatic mouse model *in vivo* [73]. Comparable findings were reported in primarily apoptosis resistant colon carcinoma cells (HCT116) harbouring an activating mutation in the *PIK3CA* gene, which yields a constitutive active form of the catalytic α-subunit of the PI3K (phosphatidylinositide 3-kinase). In this context, non-apoptotic caspase-8-mediated cleavage of ROCK1 (rho-associated, coiled coil containing protein kinase-1) caused a change in cellular morphology towards an amoeboid shape and was associated with increased invasiveness *in vitro* [74].

Recent studies also reported a role for TRAIL in immunity, e.g., in context of *Streptococcus pneumoniae* infection. In this context, soluble TRAIL induces apoptosis of alveolar macrophages, thereby limiting bacterial replication and restricting the inflammatory response [75]. In contrast to this beneficial role of TRAIL in a model of pulmonary infection, another group reported that TRAIL-promotes disease development in animal models of pulmonary arterial hypertension [76]. The role for TRAIL in host immunity has recently been reviewed [77].

#### 4.2.2. Pathways of TRAIL-Receptor Signalling

While TRAIL-R4 is restricted to non-apoptotic pathways, TRAIL-R1 and TRAIL-R2 can trigger both, apoptotic and non-apoptotic pathways. To date it is poorly understood why humans evolved two fully functional, structurally very similar receptors for TRAIL. TRAIL-R1 and TRAIL-R2 are actually often co-expressed on the same cell. However, for apoptosis induction, TRAIL-R2 seems to be more important [78], whereas the individual contribution of each receptor to non-apoptotic signalling remains to be resolved. Of note, apoptotic signalling pathways originating from TRAIL-R1 and TRAIL-R2 can be modulated by TRAIL-R3 and TRAIL-R4, as both compete for the ligand and, in case of TRAIL-R4, can also activate counteracting pro-survival pathways [79,80]. TRAIL-mediated activation of pro-survival pathways such as NFκB, protein kinase B/Akt, and MAP kinases can also occur directly through TRAIL-R1 and TRAIL-R2 [81]. Activation of these pathways is distinct: NFκB activation via TRAIL-R1 (as well as TRAIL-R2 and TRAIL-R4) involves a TRAF2-NIK-IKK complex, while TRAIL-R1-mediated JNK activation requires a TRAF2-MEKK1-MKK4 complex [64]. This indicates that non-apoptotic signals bifurcate at the level of TRAF2. Interestingly, NFκB activation is insufficient to block TRAIL-induced apoptosis in all cell types [82,83].

Apoptosis induction via TRAIL-R1 and TRAIL-R2 requires death domain-dependent recruitment of FADD and caspase-8. In contrast to Fas and TNFR1, TRAIL-R1 and TRAIL-R2 exhibit already robust DISC-formation at the plasma membrane level and thereby triggers pro-apoptotic signalling. The impact of TRAIL-receptor internalisation on signalling is discussed below.

#### 4.2.3. Membrane Trafficking and Implications on TRAIL-Receptor Signalling

In line with the observations in the TNF/TNFR1 system, internalisation of TRAIL-R1 and TRAIL-R2 is critically involved in determination of signalling outcome. But in sharp contrast to the TNF/TNFR1 system, the requirement of TRAIL-receptor internalisation for apoptosis-induction seems to be cell-type dependent. In type-I cells, stimulation with labelled ligands resulted in TRAIL-R1 and TRAIL-R2 internalisation via clathrin-dependent and -independent mechanisms with concomitant recruitment of FADD and caspase-8 [84,85]. After 2 h, caspase-dependent cleavage of components of the endocytic machinery (clathrin heavy chain and the α-subunit of the adapter protein 2) was detectable, finally terminating TRAIL-receptor endocytosis. Interestingly, this blockade in receptor internalisation did not abrogate but amplify apoptosis induction [84], and consequently rendered TRAIL-receptor internalisation as a *conditio sine qua non* for cell death induction unlikely in this experimental system. On the other hand, hepatocellular carcinoma and cholangiocarcinoma cell lines (type-II cells) displayed TRAIL-R2 internalisation upon TRAIL binding and apoptosis induction with receptor trafficking to lysosomes and subsequent release of lysosomal proteases [86]. TRAIL-R2 internalisation was dependent on a di-leucine based internalisation motif. Interference with TRAIL-R2 internalisation disturbed trafficking to the lysosome abolished apoptotic cell death. These findings indicated a pivotal role for TRAIL-R2 internalisation in terms of apoptosis induction in that system. This study also confirmed the negative findings regarding TRAIL-R1 and TRAIL-R2 internalisation in type-I cells from the study cited above.

**Figure 3:**
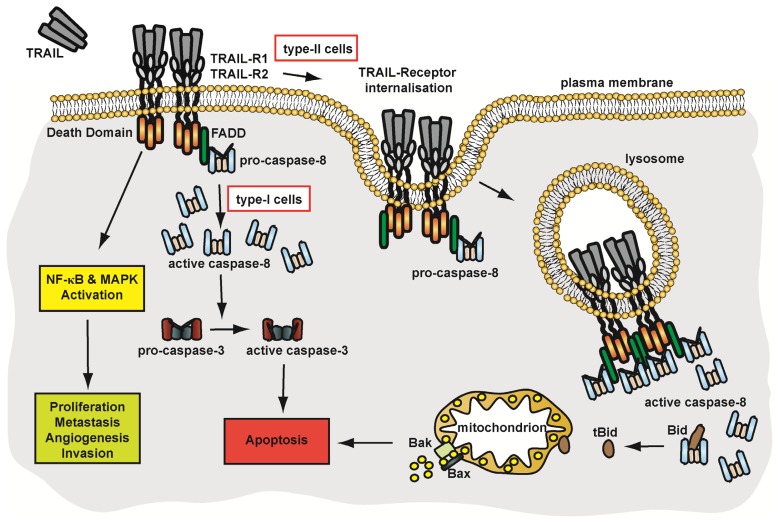
TRAIL-R1 and TRAIL-R2—mediated signalling pathways. See text for details.

In sum, TRAIL-receptor-mediated apoptosis induction is not dependent on receptor endocytosis under all circumstances. While receptor-associated DISC at the plasma membrane level is sufficient for robust apoptosis induction in type-I cells, TRAIL-mediated apoptosis induction in type-II cells may be strictly dependent on receptor internalisation. A schematic overview of TRAIL-receptor signalling is given in Figure 3.

### 4.3. The FasL/Fas System

#### 4.3.1. Structure, Physiological and Pathophysiological Roles of the FasL/Fas System

##### 4.3.1.1. Structure

The gene for FasL is located on chromosome 1q23, consists of 4 exons and spans about 8 kb of DNA [87]. The extracellular portion of FasL consists of 179 aa and contains the TNF homology domain, the characteristic structural feature of the TNF ligand family. The intracellular FasL domain (80 aa) possesses an extended proline-rich region (aa 45–65), several tyrosine phosphorylation sites and a casein kinase phosphorylation motif, which have been implicated in FasL sorting and reverse signalling [88,89]. FasL exists in two functional forms, either membrane-bound or soluble. The soluble form can be generated by proteolytic cleavage of the membrane-bound form between K129 and Q130 [90] via metalloproteinase-3 and/or metalloproteinase-7 [91,92].

The receptor Fas (FS7-associated cell surface antigen) attracted considerable interest in the field of apoptosis research since its discovery in 1989, as treatment with Fas-specific monoclonal antibodies induced robust apoptosis in cell culture. The Fas gene is located on chromosome 10q24.1 [93], spans about 26 kb of DNA and contains nine exons. Fas is a 319 aa type-I transmembrane protein, with a 157 aa extracellular and a 145 aa intracellular domain. Alternative splicing yields at least seven variants of mRNA transcripts, encoding several soluble forms of the receptor with potentially negative regulatory effects *in vivo* [94]. The extracellular domain contains three CRDs. Functionally, all are required for ligand binding, although direct contact with FasL is exclusively provided by CRD2 and CRD3 [95]. The extracellular pre-ligand assembly domain (PLAD) mediates homotypic Fas interactions and was recently mapped to aa 43–66 [96]. The C-terminal half of the intracellular domain comprises the death domain.

##### 4.3.1.2. Physiological and Pathophysiological Roles of the FasL/Fas System

Mice with FasL dysfunction phenotypically present with generalized lymphoproliferative disease (gld). Dysfunction of Fas, either caused by retroviral insertion and subsequent premature termination of transcription or a point mutation in the Fas death domain, causes lymphoproliferation (lpr) in mice [97]. These animals are furthermore prone to autoimmune diseases displaying symptoms such as lymphadenopathy, production of autoantibodies, hypergammaglobulinemia and accumulation of CD4^−^CD8^−^ T-cells [97].

In immunobiology, FasL and Fas are involved T-cell homeostasis [98] in immune privileged sites such as the eye, brain, testis, ovary, pregnant uterus and placenta [99], essentially by FasL-induced cell death of invading inflammatory cells. A recent study also reported that Fas-induced chemokine production promoted chemotaxis of phagocytes towards apoptotic cells [100], thereby influencing immune responsiveness towards dying cells. The versatile roles of Fas in the immune system are reviewed in [101]. Beyond immunology, FasL and Fas obey also functions in the cardiovascular system, in tissue differentiation and regeneration (e.g., in the neuro system and the liver), and play a dual role in cancer biology. On the one hand, pro-inflammatory Fas signalling pathways exert a pro-tumoural effect and surface expression of FasL on tumour cells mediates killing of tumour infiltrating lymphocytes, thereby establishing an immune-privileged tumour microenvironment. On the other hand, expression of FasL on T-cells and NK-cells induces cell death in Fas-bearing tumour cells. In context of malignant diseases, Fas-associated signalling was reported to enhance migration and invasion of tumour cells. Insights in underlying mechanisms came e.g., from a study using glioblastoma cells. In this model, recruitment of the Src family kinase Yes and the p85 subunit of PI3K to Fas was associated with increased expression/secretion of matrix-metalloproteases (MMPs) [102]. Beside MMP secretion, formation of actin-driven cell protrusions is considered as another crucial event in Fas-mediated invasion [103]. In colorectal cancer cells, Fas-ligation activated the cofilin pathway and initiated cortical actin remodelling with subsequent formation of membrane protrusions, finally resulting in enhanced invasiveness of the tumour cells [104]. Another recent study reported general dependency of tumour cells on constitutive Fas activation, stimulated by cancer-produced FasL [105]. Loss of Fas in mouse models of ovarian cancer and liver cancer reduced both, cancer incidence and tumour size. The tumourigenic activity was essentially attributed to a pathway involving JNK and Jun [105]. For an in-depth coverage on the role of FasL and Fas in health and disease the reader is referred to [106].

#### 4.3.2. Pathways of Fas Signalling

The soluble and the membrane-bound form of FasL bind to Fas, but only membrane-bound or artificially immobilised FasL cause robust receptor activation [107]. Mechanistically, a model of Fas activation distinguishing five stages has been proposed [108]. In a first step, ligand-binding induces formation of stable Fas micoraggregates and thereby beginning assembly of the DISC. The next steps result in formation of higher molecular Fas structures, via association with lipid rafts and coalescence into large signalling platforms. Subsequently, Fas internalisation yields high level DISC formation in the endosomal compartment. Beside FasL and Fas, the DISC consists of FADD [109] and caspase-8 [110]. Dimerization of caspase-8 is the first step in activation of this protease, but it is readily converted in its mature heterotetrameric form by autoproteolytic maturation and is subsequently released from the DISC [111]. Active caspase-8 initiates the apoptotic cascade. Caspase-8 activation at the DISC level can be inhibited by the FLIP [94]. Blocking apoptosis induction reveals non-apoptotic signalling pathways emanating from Fas such as NFκB and the MAPKs ERK (extracellular signal regulated kinase) and JNK. Activation of the NFκB pathway utilises a RIP1, FADD and caspase-8 containing complex [9]. Fas-mediated MAPK activation can either rely on caspase-dependent or independent mechanisms. For instance, JNK activation can occur by cleavage and activation of components of the JNK pathway (e.g., the MAP3K MEKK1) [112] or in the absence of caspases by recruitment of death domain-associated protein 6 (DAXX) and the apoptosis signalling kinase-1 (ASK1).

#### 4.3.3. Membrane Trafficking and Implications on Fas Signalling

Fas-associated apoptotic and non-apoptotic signalling pathways diverge at the plasma membrane level. Apoptotic signalling pathways are internalisation-dependent, whereas non-apoptotic Fas-signalling is independent of receptor internalisation. Fas internalisation occurs in an actin- and clathrin-dependent manner [113].

Using agonistic, Fas-specific antibodies, receptor internalisation is detected within the first 5–15 min. Fas internalisation after FasL binding has been reported to occur even faster [108,114]. In type-I cells, as early as 3 min after ligand binding, internalisation takes place and Fas-containing receptosomes can be isolated. Analysis of these vesicles revealed positivity for the endocytic markers Rab4, EEA1 (early endosome antigen-1) and Cathepsin D [108,115]. Even at this early time point, low levels of FADD and active caspase-8 (p43/41 and p18 fragments) were detectable. Both proteins exhibited time-dependent accumulation, peaked approximately 30 min after ligand binding and were detectable even after 3 h stimulation. Therefore, although DISC formation is initiated at the plasma membrane, assembly of components and subsequent caspase-8 activation occurs predominantly in Fas-containing intracellular compartments. This concept is not absolutely exclusive, as in certain cell populations such as lymphoblasts and T-cells, low-level DISC assembly might precede Fas internalisation [114]. In line with internalisation-dependent DISC assembly, approaches targeting the Fas internalisation motif (YDTL, aa 291–294) or knockdown of AP-2, an essential adapter protein for clathrin-mediated endocytosis, significantly inhibited DISC recruitment and apoptosis. However, non-apoptotic signalling pathways such as the activation of the NFκB and ERK pathway remained intact [108]. Pathophysiologically, especially in Fas-resistant tumour cells, activation of these pro-inflammatory and potentially pro-tumoural signalling pathways may be of clinical relevance. Beside the tyrosine-based internalisation motif, a glycosphingolipid-binding motif located in the extracellular domain of Fas was reported to be essential for clathrin-dependent internalisation [116]. Destruction of this motif abolished apoptotic Fas signalling, although receptor endocytosis occurred via an alternative route. Non-apoptotic signalling pathways remained again unaffected [116].

The prerequisite for Fas internalisation to trigger the apoptotic cell death machinery raises another interesting question: the physiologic stimulus for Fas is most likely the membrane-bound form of FasL rather than agonistic antibodies or artificially oligomerised derivatives of FasL. Does receptor internalisation also occur under these, more physiological circumstances? Indeed, Fas internalisation was observed in co-culture experiments with cells expressing a non-cleavable form of membrane-bound FasL. The degree of receptor internalisation was comparable to experiments using oligomerised soluble forms of FasL [108].

Interestingly, Fas internalisation is not the only mechanism involving membrane trafficking to ensure selective Fas signalling. Plasma-membrane distribution of this receptor is regulated by post-translational modifications. Several oxidative and non-oxidative modifications alter Fas association with lipid rafts, which is a prerequisite for robust apoptosis induction. For example, palmitoylation at the intracellular, membrane proximal residue Cys199 grants entry in lipid rafts [115,117]. Mutations of this critical cysteine residue highlight the physiological relevance of palmitoylation in Fas signalling, as formation of high-molecular Fas aggregates, Fas association with lipid rafts, FasL-induced Fas internalisation, DISC formation and apoptosis were all strongly diminished. Apart from palmitoylation, reversible oxidations such as S-glutathionylation at Cys304 [118] or S-nitrosylation of Cys199 or Cys304 [119] also led to redistribution of Fas into lipid rafts and sensitised cells to Fas-mediated killing. In contrast, extracellular N-glycosylation of Fas (Asn118 and Asn136) had no effect on DISC formation [120].

Membrane trafficking events are also important for FasL. Membrane-bound FasL has been shown to be released by exosome-like vesicles into the immunological synaptic cleft. FasL sorting to secretory lysosomes (by promoting entry into multivesicular bodies) is regulated by phosphorylation events in the proline-rich domain by Src family tyrosine kinases (Fgr, Fyn and Lyn kinases) mono-ubiquitination at lysines flanking the proline-rich domain in its cytoplasmic tail [121].

**Figure 4:**
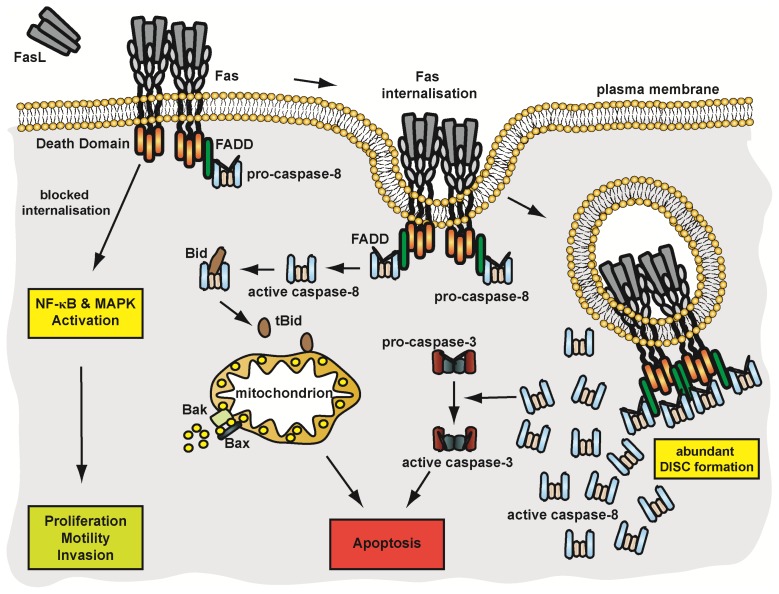
Fas—mediated signalling pathways. See text for details.

Taken together, Fas-associated, non-apoptotic signalling pathways occurs irrespective of receptor internalisation, whereas apoptosis induction critically depends on Fas recruitment in lipid rafts and efficient receptor internalisation. A schematic overview of Fas signalling is given in Figure 4.

### 4.4. The TL1A/DR3 System

#### 4.4.1. Structure, Physiological and Pathophysiological Roles of the TL1A/DR3 System

##### 4.4.1.1. Structure

TL1A was identified as ligand for DR3 in 2002 by searching expressed sequence tag databases and screening of an endothelial cDNA library [122]. The TL1A gene is located on chromosome 9p32 and consists of four coding exons. The 251 aa type-II transmembrane protein appears on SDS-PAGE at a molecular weight of 22 kDa.

The receptor DR3 was discovered in the search of proteins with sequence similarity to the intracellular parts of TNFR1 and Fas by screening cDNA libraries or using two-hybrid systems [123–126]. Almost simultaneously, a number of research groups reported identification of DR3 (at that time also known as Apo-3, TNFRSF25, TRAMP, LARD or WSL-1). The DR3 gene is located on chromosome 1p36.2, spanning about 5.04 kb of DNA and contains nine exons [125]. Alternative mRNA splicing yields at least 11 distinct DR3 isoforms, most of which are supposed to be secreted. The type-I transmembrane protein has a length of 417 aa with a 25 aa leader sequence and appears at a molecular weight of 47 kDa on SDS-PAGE. The crystal structure of DR3 has not been solved yet, but predictions based on structural modelling suggest an architecture similar to TNFR1. The extracellular domain comprises 175 aa and exhibits like TNFR1 four CRDs. Contact with the ligand TL1A is thought to occur between CRD2 and CRD3 [127]. The membrane-spanning helical domain consists of 21 aa. The intracellular domain of DR3 has a length of 197 aa and includes in the C-terminal half the DD. DR3 shares 23% of sequence similarity with Fas and 29% with TNFR1. This is in fact the highest degree of sequence similarity to TNFR1 among death receptors. Erroneously, an early report suggested interaction of DR3 with TWEAK (TNF-like weak inducer of apoptosis), another TNF-family ligand [128]. However, the conclusions were based on overexpression studies [129] and subsequent experiments could not confirm the initial findings. Additionally, identification of Fn14 (Fibroblast growth factor-inducible immediate-early response protein 14), the corresponding receptor for TWEAK, further dismounted this concept [130].

##### 4.4.1.2. Physiological and Pathophysiological Roles of the TL1A/DR3 System

DR3 expression was first recognised on peripheral blood leukocytes [125] and lymphocyte-rich tissues such as thymus and spleen. Albeit to a lesser extent, DR3 localization in the small intestine, fetal lung and fetal kidney was observed and cellular DR3 expression was reported for NK-cells, endothelial cells, macrophages and tumour cells [131–134]. TL1A enhances, like other members of the TNF family, T-cell proliferation and cytokine production. Additionally, TL1A-DR3 interactions exhibit prominent effects on T-cell polarization, T-cell homeostasis and T-cell-mediated immune response against bacteria and viruses [135]. For further information the reader is referred to [136].

Consistent with the important role of TL1A and DR3 in T-cells, a variety of T-cell dependent autoimmune diseases have been found to be driven by this receptor-ligand pair. Especially, the TL1A-mediated expansion of effector T-cells at inflammation sites and allergic diseases in the lung as well as the gastrointestinal tract has emerged from these studies [136]. DR3- [137] or TL1A-deficient [122] mouse models highlighted the importance of TL1A-DR3 interaction in experimental autoimmune encephalitis (EAE) [138,139], collagen- and antigen-induced arthritis [140] as well as pathological T-cell responses in inflammatory bowel disease [141–144].

Beside autoimmune diseases, a role for the TL1A/DR3 system in atherosclerosis [133] and tumourigenesis [145,146] has been reported. In the chemoresistant human pancreatic cancer cell line AsPC-1, DR3-induced pro-inflammatory NFκB signalling correlated with enhanced cell proliferation and invasiveness, but the underlying molecular mechanism remained unclear [145]. However, in a study using colorectal carcinoma cells, tumour-cell associated DR3 was shown to bind to endothelial E-selectin, causing pro-inflammatory p38, Akt, ERK as well as NFκB activation and furthermore promoted DR3-dependent transendothelial migration of HT29 cells in a Boyden chamber assay [131,132].

#### 4.4.2. Pathways of DR3 Signalling

At the time of discovery, death receptor-induced cellular suicide was the best studied signalling event triggered by DR3. Ectopic expression of DR3 in HEK293 resulted in substantial loss of cell viability. Observations of membrane blebbing, DNA fragmentation and rescue by cotransfecting the viral caspase inhibitor CrmA were indicative for apoptotic cells death [123]. Beside cell death induction, DR3-mediated activation of the pro-inflammatory NFκB pathway was recognised, pointing to ignition of distinct signalling pathways depending on the cellular context of DR3 activation.

First attempts to analyse the intracellular receptor signalling complex came from yeast-two-hybrid analysis, reporting interaction of DR3 with TRADD [126], a molecule also critically involved in TNFR1 signalling. In a transient system, DR3 interaction with TRADD, TRAF2, FADD and caspase-8 was reported [147]. Immunprecipitation experiments in endogenously DR3-expressing TF-1 cells, confirmed receptor association of TRADD and TRAF2 and additionally detected RIP1, whereas FADD and caspase-8 were not detected [148].

TL1A binding to DR3 resulted in activation of ERK, JNK, p38 and NFκB signalling pathways. In TF-1 cells, apoptosis induction was only detected in the presence of sensitising agents such as cycloheximide or pharmacological inhibition of the antiapoptotic NFκB pathway. In both cases, sensitization towards apoptosis was essentially traced back to impaired TL1A-induced upregulation of the antiapoptotic protein cIAP2, a direct NFκB target gene [148]. A recent study confirmed a crucial role for TRADD in DR3 signalling, as the proliferative response of CD4 and CD8 T-cells was critically dependent on TRADD and significantly diminished in T-cells from TRADD-deficient mice [149].

#### 4.4.3. Membrane Trafficking and Implications on DR3 Signalling

To date, the effects of DR3 membrane trafficking and/or compartimentalization on signalling processes emanating from this receptor are not understood and therefore remain for the most part speculative. Directly underneath the putative transmembrane domain, DR3 reveals several tyrosine residues, although no canonical tyrosine-based internalisation motif (e.g., YXXΦ, where Φ is a bulky hydrophobic amino acid) for clathrin-mediated endocytosis is observed at the first glance. However, a recent study demonstrated existence of YXXXΦ-based internalisation motifs [150]. In fact, DR3 harbors a YXXXΦ motif in the intracellular domain (amino acids 224–228), that has not yet been characterized in this respect. It is therefore tempting to speculate that, analogue to TNFR1, DR3 internalisation upon ligand binding and subsequent compartimentalization of DR3 in the receptosome constitutes a mechanism to coordinate signalling events. Studies addressing these issues are ongoing in our laboratory.

## 5. Conclusions and Perspectives

In the light of the findings discussed, early concepts reducing receptor internalisation as a simple shut-off mechanism for receptor-associated signalling events nowadays fall way too short. In context of TNFR1 and Fas, receptor internalisation after ligand binding constitutes a key mechanism to ensure signal specificity of these multipurpose receptors. Internalisation after ligand binding is indispensable for robust apoptosis induction, as efficient assembly of the DISC primarily takes place in an intracellular, death-receptor containing compartment. However, non-apoptotic signalling pathways, such as NFκB or MAPK activation, occur already at the plasma membrane level and are independent from cellular receptor uptake. That points to an elegant, spatiotemporal regulation of TNFR1 and Fas signalling. To date, none of these aspects has been sufficiently addressed for the closest relative of TNFR1, DR3.

For TRAIL-R1 and TRAIL-R2, dependency of apoptosis-induction on receptor internalisation differs between cell types. More work is needed to elucidate and functionally characterise pathways involved in internalisation of this receptor-ligand system, especially as TRAIL-receptor internalisation is apparently not exclusively dependent on classical clathrin-mediated endocytosis.

However, the necessity to control signalling outcome by compartment-restricted pathway activation comes at a price. Adenoviruses evolved a sophisticated strategy to ensure survival in infected cells and interfere with TNFR1 internalisation via the viral protein 14.7K, thereby protecting against TNF-induced cell death, a deleterious event for the virus [57]. On the other side, spatial segregation of signalling events also allows selective interventions in context of therapeutic strategies.

An in-depth understanding of receptor signalling regulation, whether by internalisation or other mechanisms, lays the ground for target-restricted therapeutic interventions in a variety of human diseases including malignancy, immunity towards infections and autoimmune processes.
